# Water Resource Recovery Facilities Meet Low‐Level Mercury Limits by Controlling Effluent Suspended Solids

**DOI:** 10.1002/wer.70158

**Published:** 2025-08-06

**Authors:** Geordee Spilkia, Scott Kyser, Adrian T. Hanson, Kelsey Hogan, Nathan W. Johnson

**Affiliations:** ^1^ Water Resources Science Graduate Program University of Minnesota St. Paul Minnesota USA; ^2^ Minnesota Pollution Control Agency St. Paul Minnesota USA; ^3^ Department of Civil Engineering University of Minnesota Duluth Duluth Minnesota USA

**Keywords:** dissolved organic matter, low‐level limits, mercury, metals, municipal wastewater, particulate matter control, tertiary filtration, TSS removal

## Abstract

Hundreds of water resource recovery facilities (WRRFs) in North America have received low‐level mercury effluent limits (< 2 ng/L). Although mercury binding to dissolved organic matter (DOM) and particulate matter in natural environments is well understood, guidance about low‐level mercury removal at WRRFs is lacking. We collected samples of filter‐passing and particulate mercury at 16 WRRFs with a variety of secondary and tertiary particle‐control technologies. Particulate mercury in WRRF effluent was covariate with total suspended solids (TSS) at concentrations ranging from < 0.2 to 15 ng/L. Filter‐passing (< 0.45 μm) mercury in WRRF effluent was mostly bound to DOM and was typically between 0.3 and 0.8 ng/L. Thermodynamic modeling and sulfur quantities in wastewater TSS and DOM point to a consistent quantity of filter‐passing Hg that cannot be removed by typical wastewater technologies and necessitates effective particulate removal to meet low‐level mercury limits.

## Summary

- Across a variety WRRF, TSS removal to < 1–3 mg/L predicted Hg removal to < 2 ng/L.
- Persistent, DOM‐associated Hg comprises a significant fraction of allotted Hg for facilities with low Hg limits.
- Municipal facilities with industrial influence may carry more Hg through to effluent due to abundant dissolved organics.
- To meet low Hg limits, engineers should prioritize particle removal technologies capable of controlling TSS to < 2 mg/L.

## Graphical abstract

In municipal wastewater effluent, the quantity of total mercury (filter passing + particulate forms) is determined primarily by the final particulate removal technologies before discharge. The effectiveness of this particle removal determines the ability of a facility to consistently meet low‐level permit limits.

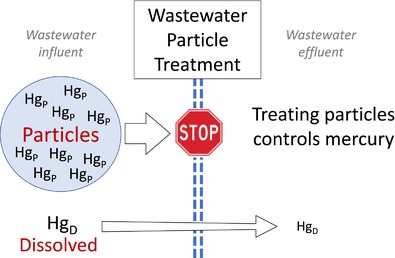

## 1 Introduction

In recognition of mercury's impacts on human health and the environment, 152 countries are either signatory or party to the international Minamata Convention, an agreement that seeks to identify and minimize anthropogenic sources of mercury (Hg) from industrial sources (Kessler 2013) as well as from Water Resource Recovery Facilities (WRRF). Mercury mass flows in WRRFs are a small portion of the global Hg budget and are often not tracked because WRRFs receive and remove aqueous Hg in waste streams but do not add new Hg to the global budget (Munthe et al. 2019; Outridge et al. 2018). However, Hg discharges from WRRFs play a significant role in aquatic systems (Bodaly et al. 1998; Suess et al. 2020), especially under low‐flow river conditions when Hg from WRRFs comprises a large portion of Hg in surface waters and dry soils can promote Hg methylation and bioaccumulation (Azevedo et al. 2018; Paranjape and Hall 2017). Effective methods to control local, point‐source loads of Hg represent an opportunity to lower Hg exposure through fish consumption. The US federal government has promulgated a very low total Hg water surface quality standard (1.3 ng/L) for states bordering the Laurentian Great Lakes to honor international agreements and treaties with sovereign tribal nations (Environmental Protection Agency 1995a) and to protect sensitive populations with increased exposure risk (McCann 2011). Hundreds of WRRFs in this region have received low‐level (< 2 ng/L) Hg effluent limits that are much lower than in the European Union (Kern 2014) (< 70 ng/L), Switzerland (Swiss Federal Council 1998), and other US states (< 10 ng/L) (Environmental Protection Agency 1995a). To comply with stringent Hg effluent limits and maintain high quality in valuable waters, an urgent need exists to document methodologies that can remove Hg from wastewater to concentrations < 2 ng/L.

A robust body of literature has evaluated mass flows of Hg and Hg removal from municipal wastewater at individual facilities and for individual technologies (Bodaly et al. 1998; Suess et al. 2020; Balogh and Liang 1995; Stoichev et al. 2009; Mao et al. 2016; Hsu and Sedlak 2003; Beutel et al. 2019; Wu et al. 2016). During biological wastewater treatment, the majority of Hg mass becomes associated with organic particulates (Mao et al. 2016; Hsu and Sedlak 2003) and is removed from water through sedimentation (Suess et al. 2020; Hsu and Sedlak 2003). Conventional secondary treatment effectively removes 88%–99% of Hg from water to levels typically ranging from 2 to 20 ng/L (Bodaly et al. 1998; Balogh and Liang 1995; Stoichev et al. 2009) but cannot consistently treat Hg to < 2 ng/L. At bench and pilot scales, tertiary treatment technologies (Beutel et al. 2019; Wu et al. 2016; Urgun‐Demirtas et al. 2012; Huang et al. 2015) have removed Hg from municipal wastewater to < 2 ng/L, though many of these findings have not been verified at full‐scale facilities or in the context of varying influent water characteristics. Because Hg binds strongly to reduced sulfur in particulates, dissolved organic matter, and inorganic ions, the partitioning strength and abundance of sulfur in these complexing phases in wastewater effluent could be important for the design of tertiary treatment aimed at very low‐level Hg removal (Pham et al. 2014; Poulin et al. 2017; Dong et al. 2011; Skyllberg 2008). Despite the widespread need for mercury removal from municipal wastewater and an abundance of insights to Hg mass flows and partitioning, no published studies have developed a Hg treatment theory or systematically evaluated a breadth of full‐scale treatment technologies for low‐level Hg limit compliance at WRRFs.

This study seeks to document the removal of Hg at WRRF subject to strict Hg effluent limits and elucidate the factors influencing how Hg partitions among particulates, dissolved organic matter, and inorganic complexes in municipal wastewater. We combine observations of filter‐passing and particulate‐associated Hg at full‐scale facilities, characterization of dissolved organic matter and particulate matter, and Hg complexation modeling to explain how Hg is carried through diverse treatment approaches employed at WRRFs and recommend strategies to successfully meet low‐level Hg limits.

## 2 Methods

### 2.1 Conceptual Approach and Wastewater Treatment Facility Selection

This study made observations at 16 different full‐scale, operational WRRFs (Table 1) selected to encompass a variety of secondary treatment approaches and tertiary particle control strategies. The facilities vary in size and source water characteristics. Most of these WRRFs are subject to an effluent Hg limit of 1.8 ng/L due to their discharges into the Laurentian Great Lakes watershed, whereas others are subject to a 10 ng/L effluent limit. Three facilities have significant industrial wastewater contributions (paper mill and dairy products), but none have known major Hg sources. Every facility has a Hg management plan in their permit that requires them to identify and minimize sources of Hg loading to the WRRF. All of the facilities report Hg concentrations in their biosolids that are below the applicable federal limits for land application.

**TABLE 1:** Municipal WRRFs included in the study, organized by tertiary treatment and secondary treatment. Source water characteristics, industrial contributions, and effluent limits are included for context. All facilities are located in the state of Minnesota. Facilities with 1.8 ng/L effluent Hg limit are in the Laurentian Great Lakes Basin.

Tertiary category	Tertiary technology	Secondary technology	Design flow (MGD)	Source water	% Industrial flow	Effluent Hg limit^a^ (ng/L)	Facility ID^b^
Granular media	Dual media filter	Activated sludge	0.54	Groundwater	< 1%	10	1
	Dual media filter	Activated sludge	3.1	Groundwater	25%^f^	10	2
	Mono media filter^c^	Activated sludge	0.9	Mine pit	< 1%	1.8	3
	Dual media filter	Activated sludge	0.91	Mine pit	< 1%	1.8	4
	Dual media filter	Activated sludge	1.6	Lake superior	< 1%	1.8	5
	Dual media filter	Activated sludge	4.3	Mine pit	< 1%	1.8	6
	Dual media filter	Activated sludge (pure oxygen)	48	Lake superior	50%^e^	1.8	7
Membrane	Membrane bioreactor (0.01 μm)	Membrane bioreactor	0.69	Groundwater	< 1%	1.8	8
	Membrane bioreactor (0.01 μm)	Membrane bioreactor	2.0	Groundwater	< 1%	10	9
	Rotating disk filter (10 μm)^d^	Sequencing batch	2.5	Mine pit	< 1%	1.8	10
Clarifier	Contact clarifier	Trickling filter	0.92	Lake superior	< 1%	1.8	11
	Polishing pond	Activated sludge	15	River water	50%^e^	10	12
None	None	Activated sludge	0.5	Groundwater	< 1%	1.8	13
	None	Trickling filter	0.68	River water	< 1%	1.8	14
	None	Pond (facultative lagoon)	0.21	Mine pit	< 1%	1.8	15
	None	Pond (facultative lagoon)	0.04	Lake superior	< 1%	1.8	16

Abbreviation: MGD, million gallons per day.

^a^ Regional Hg_T_ discharge limits for non–Great Lakes (10 ng/L) and Great Lakes (1.8 ng/L) watersheds.

^b^ Facility ID refers to raw data tables (Table S4) in Supporting Information.

^c^ Moving bed upflow, deep bed, and granular monomedia filter.

^d^ 10‐um cutoff membrane filter.

^e^ Industrial source is paper mill wastewater.

^f^ Industrial source is dairy product wastewater.

Facilities in the study employ tertiary particle control technologies including granular media (seven facilities), membrane filtration (three facilities, 0.01 and 10 μm), and gravity clarification (two facilities). Most have conventional activated sludge (CAS) as secondary treatment; two WRRFs have shallow, rock‐media trickling filters; one has a sequencing batch reactor; and two have membrane bioreactors (MBR) that also function as tertiary particle control. Two mechanical WRRFs in the study have no tertiary treatment and two pond systems (facultative lagoons, considered a secondary technology) are not mechanical and discharge intermittently, only during the fall and spring.

### 2.2 Sample Collection and Preservation

Grab samples were collected from each WRRF at the influent and following secondary treatment (Figure S1). Samples were collected primarily in fall and winter seasons with temperatures ranging from 10°C to 20°C. In facilities with tertiary particle control, a grab sample was also collected following tertiary treatment, prior to disinfection (if present). Samples for Hg analysis were obtained directly out of pipes or by using an acid‐washed high‐density polyethylene dipper, twice rinsed with wastewater before sample collection. Water was placed in new, Hg‐free, double‐bagged polyethylene terephthalate glycol (PETG) bottles following a double rinse with sample. All samples collected for Hg analysis followed the clean hands protocol as described in EPA Method 1669 (Environmental Protection Agency 1995b). Blanks and duplicates were handled identically to the wastewater samples. A field blank was collected once per field sampling day using MilliQ (> 18 MΩ) water from the laboratory with a Hg concentration below detection limits (a mean of 0.1 ng/L and standard deviation of 0.08 ng/L; Table S1). A duplicate was collected once per field sampling day. Most WRRFs were also sampled for particulate matter (PM) and dissolved organic matter (DOM) isolation and analysis. Samples for DOM and PM were grabs from the influent and effluent into new, 5‐gal low‐density polyethylene (LDPE) buckets rinsed three times with sample. A clean 3‐gal bucket with a nylon rope cord was used for collection when samples could not be collected directly into containers.

Samples for dissolved organic carbon (DOC), specific ultraviolet absorbance (SUVA), dissolved mercury (Hg_D_), cation, and anion analysis were filtered through 0.45‐μm polyethersulfone filters (Flip‐Mates, Environmental Express, EW‐35202‐09) via vacuum pump within 24 h of collection. A well‐homogenized, split sample was used to quantify unfiltered mercury (Hg_T_). For this study, an effectively dissolved mercury fraction, Hg_D_, was defined as all mercury passing a 0.45‐μm filter.

Both Hg_T_ and Hg_D_ samples were preserved with 0.5% Trace Metal‐HCl (v/v; 34%–37%, Fisher Scientific) and stored in the dark in new, double‐bagged PETG bottles (ThermoFisher). Absorbance at 254 nm was quantified on filtered samples within 24 h of filtration. Dissolved organic carbon samples were preserved with 0.1% HCl (v/v; HCl 37%, Sigma) and stored in acid‐washed and combusted amber glass vials. Cation samples were preserved with 0.5% HNO_3_ (v/v; nitric acid 70%, Fisher) and stored in 15‐mL polypropylene centrifuge tubes. Borosilicate glass bottles to preserve samples for inorganic sulfide analysis were preloaded with 0.1% (*v*:*v*) 1 N ZnAc, filled to the brim with unfiltered water, and sealed with 1‐cm‐thick butyl rubber stoppers. Samples for anion analysis were stored in new PETG bottles at 4°C until analysis. Samples for TSS were collected from the same grab as THg samples and retained on a 1.2‐μm glass fiber filter and dried at 105°C overnight. Bulk water was processed for DOM isolation with Bond Elut PPL cartridges (Agilent, 12255002) following the method described in Dittmar et al. (2008). Detailed DOM isolation methods are provided in Text S1. PM was obtained by filtering samples through a 1.2‐μm, polycarbonate membrane filter (IsoporeTM, RTTP04700). The particulates retained on the filter were scraped into a combusted glass vial with a metal spatula and freeze‐dried. Thus, both PM and TSS are defined in our observations as particulates retained on a 1.2‐μm filters: TSS measurements were obtained and associated with Hg grab samples at all facilities, and PM measurements were associated with DOM isolate grab samples at a subset of facilities.

### 2.3 Laboratory Sample Analysis

Total Suspended Solids and Volatile Suspended Solids (VSS) were measured following Standard Methods 2540D (Rice et al. 2012). Well‐homogenized, replicate samples were vacuum filtered through a precombusted and weighed 1.5‐μm glass‐fiber filter (Whatman, 934‐AH) to collect particulates. Materials from DOM and PM isolates were measured for elemental composition (CNS) using a Vario EL Cube. The samples were weighed into tin capsules and dosed with 38 mL/min O_2_ for 90 s. The CO_2_ trap is heated to 240°C for 90 s, and the SO_2_ trap is heated to 260°C for 150 s. DOC was analyzed using the high‐temperature combustion method described in Standard Methods 5310B (Rice et al. 2012) in a total organic carbon analyzer instrument (TOC‐Shimadzu). SUVA analysis followed EPA Method 415.3 (Potter and Wimsatt 2012) using a 1‐cm quartz cell. Absorbance measurements were made on a filtered, unpreserved sample using a Hach DR 5000 Spectrophotometer. All SUVA quantities were corrected for the presence of iron using the methods outlined in Weishaar et al. (2003). Anions were measured by ion chromatography (IC) using a Dionex Integrion HPIC according to EPA Method 300.0 (Pfaff 1993). Cations, including iron, were analyzed by inductively coupled plasma atomic emission spectroscopy (ICP‐AES) using an iCAP 7600 (Dahlquist and Knoll 1978). Dissolved sulfide was measured using the methylene blue method (Rice et al. 2012) in a 1‐cm cuvette and had a detection limit of 0.016 mg/L. Additional method details for TSS and VSS are provided in Text S1 and Table S2.

Hg analysis was completed in accordance with EPA Method 1631 (Environmental Protection Agency 2002). Mercury in filtered and unfiltered water was determined using bromine monochloride oxidation followed by stannous chloride reduction, dual gold amalgamation, and quantification on a Brooks Rand MERX‐T system. Methyl mercury was quantified on select samples using an isotopically enriched internal standard following distillation (Hintelmann and Evans 1997). A few measurements on select influent and effluent samples showed that methyl mercury concentration was, on average, 0.06 ng/L for effluent and secondary effluent samples (*n* = 9) and 0.5 ng/L for influent samples (*n* = 7). These methyl mercury quantities comprise less than 5%–10% of total mercury, and methyl mercury was not considered a major contributor to mercury mass flow in the largely aerobic wastewater conditions included in this study. QA/QC data for Hg analysis, including results of replicate sample grabs, replicate lab analyses, detection limits, and recovery of reference material is included in Table S1 and Text S1.

### 2.4 Data and Statistical Analysis

In this study, total mercury (Hg_T_) is defined as the concentration (ng/L) of all Hg species in an unfiltered water sample. Dissolved mercury (Hg_D_) is defined as the concentration (ng/L) of all Hg species in a water sample that passed a 0.45‐μm filter. Particulate Hg (Hg_P_) is defined as the concentration (ng/L) of all Hg species in colloids or on particles greater than 0.45 μm and was calculated as the difference between the homogenized and split Hg_T_ and Hg_D_ measurements for each sample. To account for variability in the wastewater matrix, three independent replicate grab samples from the same day at the same facility location were collected and analyzed separately for Hg and TSS (Text S1). The average of replicate lab analyses for a single grab sample was used to calculate the average Hg_T_ and Hg_D_ for the wastewater on a particular date and WRRF location.

The solid‐phase mass fractions of sulfur and carbon: C_S,DOM_, C_C,DOM_ and C_S,PM_, C_C,PM_, are reported for the DOM retained on PPL cartridges and PM retained on a 1.2‐μm filter, respectively. The volumetric concentration of sulfur per liter of water in dissolved organic matter (DOM_S_) was calculated by dividing the DOC concentration by the carbon fraction in the PPL isolate (C_C,DOM_) and multiplying by the sulfur fraction in the PPL isolate C_S,DOM_. The volumetric concentration of sulfur per liter of water in the particulate phase (PM_S_) was found by multiplying TSS by C_S,PM_. The solid‐phase concentration of Hg per milligram of TSS (C_Hg,TSS_) was calculated by normalizing the Hg_P_ by TSS. The effective partition coefficient, K_D_, was found as the ratio of C_Hg,TSS_ to Hg_D_ (L/kg) and is reported on a log scale.

Historic data for Hg_T_ and TSS in wastewater were retrieved from a database comprised of permit compliance data over the past 10 years from the WRRFs included in the study. These historic measurements were quantified for Hg_T_ and TSS using identical lab methods to those used in this study and encompass a broader set of wastewater conditions encountered by each of the MWTTP particle removal technologies. Historic data were compared to observations from this study to understand the representativeness of the conditions we encountered.

To compare mean concentrations at different facility locations, the significance of differences was evaluated with ANOVA after a log transformation. Single factor ANOVA was used to evaluate differences in Hg_T_, Hg_D_, TSS, and DOC among facility locations. Regression analysis (after log transformation) was used to evaluate the significance of relationships between Hg_D_, Hg_P_, and other independent variables.

### 2.5 Mercury Complexation Modeling

The distribution of Hg_T_ between solid‐phase (Hg_P_), dissolved organic ligands (Hg_D_), and dissolved inorganic ligands (also a component of Hg_D_) was interpreted with complexation modeling. Bulk elemental measurements of C_S,DOM_ and C_S,PM_ were similar in magnitude to the quantities of sulfur found in prior measurements of organic matter in natural systems (Poulin et al. 2017). Measurements of sulfur in DOM and TSS in this study were used to infer the concentration of the most abundant organic and inorganic Hg species (Poulin et al. 2017; Skyllberg 2008). These were included in a complexation model to interpret the difference between Hg bound to PM_S_, Hg bound to DOM_S_, and Hg bound to inorganic sulfide (Skyllberg 2008). Additional information and assumed thermodynamic constants for complexation modeling are provided in Table S3 and Text S2. The calculated quantities of DOM‐bound Hg and inorganic‐bound Hg sulfide were associated with the filter‐passing phase, and the quantity of PM‐bound Hg was associated with the particulate (nonfilter passing) phase.

## 3 Results and Discussion

### 3.1 Mercury and TSS Removal in Each Stage of Treatment

**FIGURE 1:**
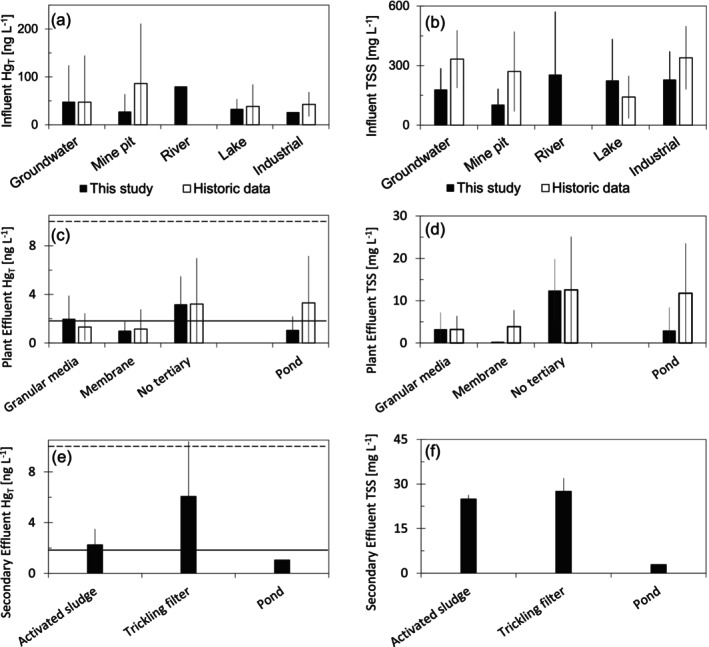
Total mercury (left column) and TSS (right column) in data from the current study (filled symbols) and historic database (open symbols) for WRRFs included in this study. Mercury and TSS are not available in the historic database for secondary effluent. Hg_T_ discharge limits for non–Great Lakes (10 ng/L, dashed) and Great Lakes (1.8 ng/L, solid) watersheds in the state of Minnesota are shown as horizontal lines. Error bars represent ± 1 SD of measurements in each category.

The influent water at municipal wastewater facilities, in both historic data and measurements made in this study, typically contained Hg_T_ between 15 and 150 ng/L and TSS between 50 and 300 mg/L (Figure 1a,b and Table S4). There was no clear relation of influent Hg_T_ or TSS to source water category, and three facilities with industrial influence (two paper and one dairy) did not have greater Hg_T_ or TSS in influent water or a discernibly larger fraction of Hg_D_ in influent water compared to the other facilities in the study (Table S4). No discernible pattern was present in replicate samples collected from the same facility during different seasons or temperatures. Influent wastewater contained far higher THg than background water in the region (median 2.2 ng/L; Table S5).

Effluent Hg_T_ at wastewater facilities was always below 10 ng/L. Facilities with granular media or membrane‐based particulate removal consistently removed Hg_T_ to concentrations lower than those at facilities without tertiary particle control (Figure 1c; *p* = 0.05 this study; *p* < 0.01 historic), typically to less than 2 ng/L. Effluent wastewater had TSS between 0.5 and 12 mg/L and was lower in facilities with granular media or membrane filtration compared to those without tertiary particle control (Figure 1d, *p* < 0.01 this study; *p* = 0.08 historic). The measurements from this study are consistent with historic data for WRRFs with tertiary particle control showing average effluent Hg_T_ concentrations between 0.7 and 5 ng/L and TSS concentrations between 1 and 8 mg/L. WRRFs with membrane‐based tertiary filtration (MBR and rotating disk filters) removed Hg_T_ to below 1 ng/L and TSS to below 2 mg/L (Figure 1c,d and Table S4).

Secondary treatment at mechanical facilities (both activated sludge and trickling filters) removed 80%–90% of influent Hg_T_ and TSS (Figure 1e,f). Following secondary treatment, Hg_T_ concentrations ranged between 1.0 and 13.2 ng/L, and TSS ranged between 20 and 30 mg/L. Attached growth secondary treatment (trickling filter) was less effective than traditional activated sludge in terms of Hg removal and similarly effective for TSS removal, consistent with other observations of poor colloid removal and a smaller particle size distribution for trickling filter effluent (Marquet et al. 2007; García‐Mesa et al. 2010). Secondary effluent Hg_T_ and TSS concentration—prior to tertiary solids removal—was not available in historic data for facilities with tertiary particle control.

At the pond systems included in this study, effluent water samples, on average, contained less than 2 ng/L Hg_T_. However, in the historic data, ponds have a wide range of Hg_T_ and TSS in effluent water (< 0.5 to > 8 ng Hg_T_/L; Figure 1c), and it is likely the samples collected and analyzed in this study are not representative of Hg removal during pond or lagoon treatment conditions. The required 150+ day pond hydraulic retention time allows sufficient time for significant denitrification to occur (Ferrara and Avci 1982) and for wastewater‐associated particulates to settle. These ponds behave analogously to shallow lake ecosystems and include interactions with the atmosphere, algal growth, and other DOM‐ and POM‐altering processes that create different Hg sources and Hg removal mechanisms compared to mechanical facilities (Bawiec and Pawęska 2020). This biological complexity likely contributes to inconsistent net Hg removal at ponds.

### 3.2 Dissolved and Particulate Mercury Removal

**FIGURE 2:**
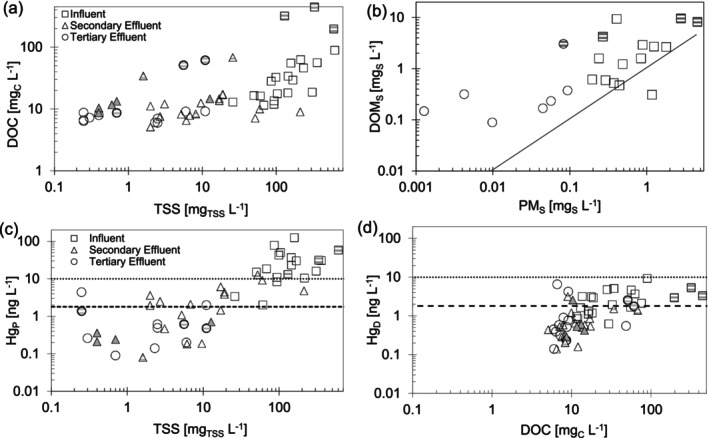
(a) Dissolved organic carbon and TSS in WRRFs included in this study for influent (squares), secondary effluent (triangles), and tertiary effluent (circles) wastewater samples. (b) Dissolved (< 0.45 μm) and particulate (> 0.45 μm) sulfur concentrations, (c) particulate Hg_P_ (> 0.45 μm) versus TSS, and (d) dissolved Hg_D_ (< 0.45 μm) versus DOC in wastewater from WRRFs. Samples from plants with DOM‐rich industrial influence are indicated with gray dashes. Pond effluent is indicated with gray triangles. Regional Hg_T_ discharge limits for non–Great Lakes (10 ng/L) and Great Lakes (1.8 ng/L) watersheds are shown as horizontal lines.

Dissolved organics (as DOC) were present in the influent of nonindustrial wastewater at concentrations ranging from 15 to 60 mg/L and were significantly lowered between influent and both secondary effluent and tertiary effluent (Figure 2a, *p* < 0.01 for influent vs. secondary and tertiary effluent combined). Although tertiary and secondary technologies sometimes removed TSS to < 0.5 mg/L, a limit of DOC removal was reached at 5 to 8 mg/L (Figure 2a), a practical limit of DOC removal from wastewater consistent with values reported elsewhere (Imai et al. 2002; Krasner et al. 2009).

Consistent with observations of TSS removal to very low levels, Hg_P_ decreased by a factor of 10 or more during secondary treatment (Figure 2c and Table S4), and, in most cases, Hg_P_ was further reduced through tertiary treatment to between < 0.2 and 2 ng/L. A regression of logHg_P_ versus logTSS for influent and secondary effluent data (Figure 2c, *p* < 0.001) as well as secondary effluent and tertiary effluent data (Figure 2c, *p* = 0.014, *p* = 0.004 with industrial influence removed) suggests that TSS is related to Hg_P_ throughout WWRFs. In paired samples (on the same day), Hg_P_ in effluent wastewater was, on average, 95% lower than Hg_P_ in influent wastewater and, on average, > 99% lower than influent wastewater in facilities with membrane filtration. Consistent with observations of DOC removal, Hg_D_ concentrations were highest in influent wastewater (0.9–10 ng/L) and decreased by a factor of 3–5 (to 0.3–0.8 ng/L) in the secondary and tertiary effluent of facilities without industrial influence (Figure 2d). A regression for logHg_D_ versus logDOC for influent and secondary effluent data (Figure 2d, *p* < 0.001) suggests that Hg_D_ is related to DOC in secondary treatment, although a regression for secondary effluent and tertiary effluent data (Figure 2d, *p* = 0.09, *p* = 0.4 with industrial influence removed) suggests that DOC and Hg_D_ are not clearly related in tertiary treatment, particularly in facilities without industrial influence. The removal of Hg_D_ from influent to effluent was much smaller (on average, 58%) than the removal of Hg_P_, an observation that is consistent with the persistence of DOC in wastewater effluent.

Some facilities with industrial sources had influent wastewater with DOC in excess of 200 mg/L, though neither Hg_D_ nor Hg_T_ was higher in the influent of these facilities. At analogous points in the treatment process, Hg_P_ was not consistently higher or lower in wastewater from industrial facilities compared to those without industrial inputs. Some secondary and tertiary effluent wastewater at facilities with industrial sources had DOC in excess of 40 mg/L, and, for effluent and postsecondary samples, both Hg_D_ and DOC were consistently higher in wastewater from industrial facilities compared to those without industrial inputs. Two facilities, one with industrial influence and one MBR, had Hg_D_ in effluent water that exceeded 2.0 ng/L.

The observation that concentrations of DOC were similar in the secondary effluent and tertiary effluent is consistent with the fact that removal of DOC to 5–8 mg/L is sufficient for WRRFs to meet typical Biochemical Oxygen Demand (BOD) limits (10 to 20 mg_BOD_/L). Although drinking water treatment often targets total organic carbon removal to < 2 mg/L (Sillanpää et al. 2018), WRRFs typically have little motivation to remove DOC to very low levels. In contrast, all WRRFs are required to remove TSS to low levels to comply with effluent limits for TSS and other particle‐associated contaminants (e.g., phosphorus and pathogens).

**FIGURE 3:**
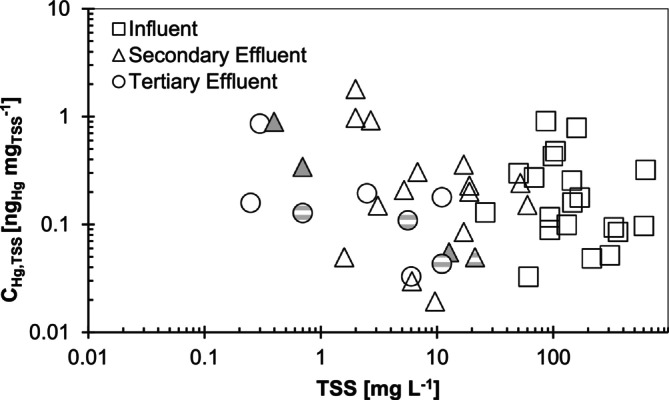
C_Hg_,_TSS_ versus TSS in WRRF samples. Samples from facilities with DOM‐rich industrial influence are indicated with gray dashes. Pond effluent samples are indicated with gray triangles.

The solid‐phase concentration of particulate‐associated mercury, C_Hg,TSS_ (Hg per mg of TSS), was variable (0.02–1.0 ng_Hg_/mg_TSS_) and did not change significantly from influent to secondary or tertiary effluent (Figure 3). There was also no clear difference in effluent C_Hg,TSS_ among the different secondary or tertiary treatment approaches. Because Hg_D_ does decrease as wastewater treatment progresses, particulate Hg may not be at equilibrium with Hg_D_. The persistently high C_Hg,TSS_ in wastewater effluent could be due to a lack of time for equilibrium with decreasing Hg_D_ during the typical 4‐ to 12‐h residence time of mechanical WRRFs or due to a re‐equilibration of Hg among the solids that remain suspended in the facility effluent. Alternatively, Hg could be preferentially adsorbing to portions of the TSS pool that are less easily broken down biologically or less able to be removed through sedimentation. On average, the effective partition coefficient (*K*_*D*_) increased from influent to effluent as the DOC:TSS ratio increased (Figure S2, *p* < 0.05 for regression of logK_D_ vs. log DOC:TSS, excluding industrial facilities), illustrating the relatively greater Hg quantities on the solid phase compared to the dissolved phase in wastewater effluent. Mechanistic explanations of partitioning are not possible with the data collected in this study, and equilibrium is unlikely in relatively rapid wastewater processes; however, the observations suggest that, as wastewater treatment progresses, solid phase Hg concentration on particulates remains consistent even as filter‐passing Hg_D_ becomes lower.

### 3.3 Dissolved and Particulate Mercury Binding Phases in Wastewater Effluent

Though TSS and DOC are widely used measurements for wastewater, the most relevant binding phases for Hg in aerobic municipal wastewater are sulfur in DOC and TSS. Therefore, a direct comparison of the quantity of S in each phase and thus a stoichiometric estimate for the quantities of binding sites in filter‐passing and particulate phases requires normalizing for the C and S content of each phase. When DOC and TSS are expressed as DOM_S_ and PM_S_, dissolved organic sulfur is typically larger than particulate sulfur (Figures 2b and S5b). Similar to DOC and TSS, there was also a limit to DOM_S_ removal of 0.1–0.3 mg_S_/L, though PM_S_ was removed to < 0.01 mg_S_/L. Dissolved sulfide (Table S4) was below the detection limit of 0.016 mg_S_/L in more than half of the effluent and postsecondary wastewater samples (average 0.036 ± 0.016 mg_S_/L for 4 samples above detection limit) and ranged from 0.03 to 0.76 mg_S_/L in influent samples (average 0.22 ± 0.23 mg_S_/L for 10 samples above detection limit). These concentrations of dissolved inorganic sulfur are well below the quantities of organic S present in DOM_S_ at analogous points in the treatment process and similar to that present in PM_S_ and are accounted for with complexation modeling later in this paper (Section 3.5).

The concentration of Hg per mass of suspended solids particles did not consistently differ among locations in the treatment facility (Figure 3), and the size of remaining particles is expected to decrease through the treatment process. The lack of a consistent increase or decrease in mercury per mass of TSS through the treatment process suggests that removing TSS mass (> 1.2 μm) by any means is likely to remove a similar amount of Hg mass, regardless of the particle size targeted or the tertiary particle removal approach. Though this study did not examine how Hg partitioning relates to effluent particle size distribution, the particle size targeted by tertiary treatment approaches included in this study is well characterized. Membrane bioreactors remove > 99% of particles larger than ~1 μM (de la Torre et al. 2013). Cloth disk filters (analogous to the 10‐μm cutoff membrane filter at one facility in this study) remove more than 80% of particles > 10 μm (Wilén et al. 2016); media filters remove 1‐ to 10‐μm fine particles with 60%–70% efficiency (Kaminski et al. 1997), trickling filters treat < 10‐μm fine particles worse than conventional secondary clarification (García‐Mesa et al. 2010), and conventional secondary clarifiers release a relatively high proportion of < 10‐μm fine particles compared to any form of tertiary filtration (Garcia‐Mesa et al. 2012). Total suspended solids are one of the most widely used wastewater design metrics, and several tertiary treatment technologies included in this study, including well‐functioning media filters, membrane disk filters, and membrane bioreactors, removed TSS to < 2 mg/L (near typical lab‐reported TSS detection limits of 1 mg/L). These were also the technologies that most effectively removed Hg_T_ to < 2 ng/L.

### 3.4 Changes to Mercury Binding Phases During Wastewater Treatment

For influent wastewater, the S content of DOM and PM was variable and did not show a clear relation to carbon (Figure S3a). For effluent wastewater, the S content of PM (C_S,PM_) was proportional to the C content of PM (C_C,PM_) (Figure S3b, *p* < 0.01, 1% molar S:C). Although the S content of effluent DOM (C_S,DOM_) was more variable compared to the S content of effluent PM (C_S,PM_), it was consistently lower than influent C_S,DOM_ and varied only between 0.5% and 1.5% molar S:C (Figure S3b). The convergence of the S content of both PM and DOM to a consistent S:C ratio in wastewater effluent may reflect a change in the nature of binding phases for Hg and is likely related to the preferential degradation of certain pools of dissolved and particulate organic matter through the wastewater treatment process (Maizel and Remucal 2017; Park et al. 2009). The net effect of these changes could reflect a uniformity of Hg binding properties in remaining effluent wastewater particles and DOM that explains the relatively consistent 0.3–0.8 ng/L Hg_D_ observed in effluent wastewater in this study.

**FIGURE 4:**
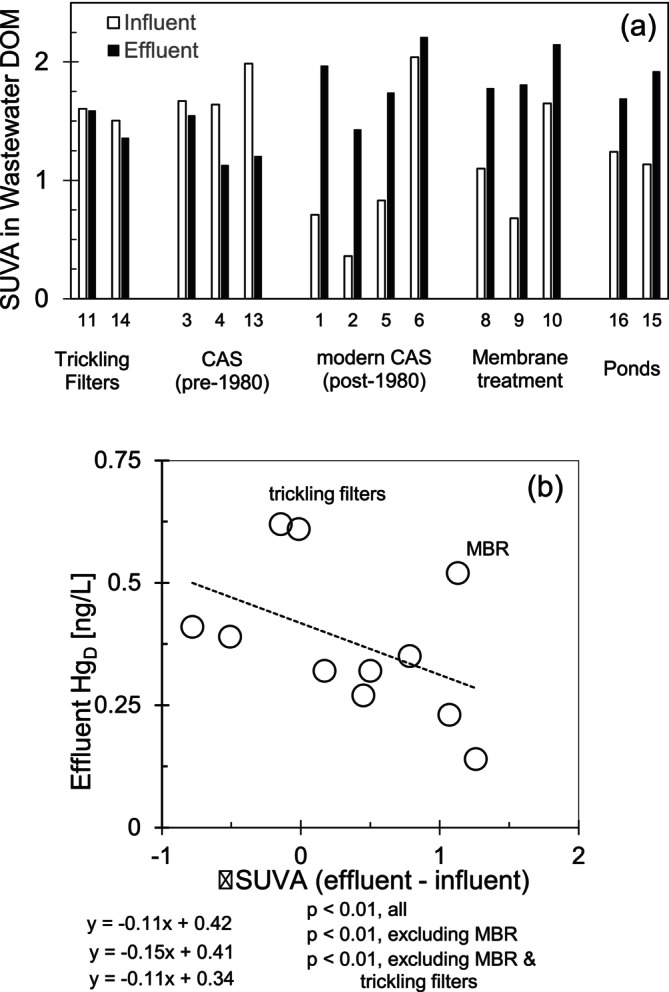
(a) SUVA in municipal wastewater influent and effluent. (b) The relation of Hg_D_ to ΔSUVA between municipal wastewater influent and effluent.

In wastewater effluent, SUVA was higher compared to the influent at most facilities (Figure 4a), especially those with more active and effective secondary treatment. This increase in SUVA is consistent with prior research that suggests the wide variety of molecular structures present in domestic wastewater dissolved organic matter shifts during treatment, becoming more recalcitrant and enriched in primarily hydrophilic organic acids with greater aromaticity after carbohydrate‐rich and aliphatic compounds have been preferentially degraded (Imai et al. 2002; Maizel and Remucal 2017). An increase in SUVA indicates an increased relative abundance of aromatic organic compounds. The facilities that did not experience an increase in SUVA between influent and effluent were the trickling filters and conventional activated sludge facilities built more than 40 years ago. Because aliphatic compounds are generally easier to biologically degrade, preferential degradation of aliphatic compounds during the activated sludge process may increase the proportion of remaining SUVA‐rich compounds, creating “weathered” DOM in wastewater effluent (Maizel and Remucal 2017; Park et al. 2009). Although some studies have found that aromatic compounds bind Hg stronger than aliphatic compounds (Wang et al. 2022), there was a consistent, if variable, decrease in Hg_D_ in effluent wastewater of facilities that had larger ΔSUVA increase (defined as the difference between effluent and influent SUVA; Figure 4b). This difference persisted, even when Hg_D_ was normalized by the quantity of DOC, though the relationship was less significant (Figure S4a). Several reasons could account for the observed decrease in Hg_D_ with increasing SUVA, and the methods we employed cannot track Hg bound to distinct types of DOM. Our interpretation is that facilities with less modern biological treatment and less chance for contact between biomass and DOM‐bound Hg, including trickling filters and old WRRF, have a smaller increase in SUVA through the facility and are less able to remove dissolved Hg_D_ to very low concentrations.

### 3.5 A Balance Between Dissolved and Particulate Hg—Relation to TSS

**FIGURE 5:**
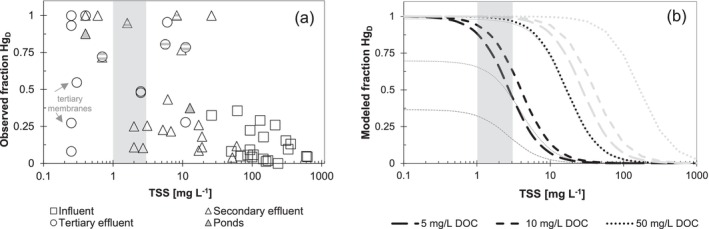
(a) Observed fraction Hg_D_ (of Hg_T_) versus TSS for wastewater samples. Samples from plants with a DOM‐rich industrial influence are highlighted with gray dashes. Pond effluent samples are indicated with gray triangles. (b) Modeled prediction of the fraction of Hg bound to filter‐passing reduced thiol groups (=Hg[RS]_2_|_DOM_) and inorganic sulfide (=HgS_2_H^−^) for different wastewater DOC concentrations. All calculations use logK_=RSH_|_DOM_ = 22.0, [H_2_S]_total_ = 10^–6.6^ M, and pH = 7.5; black lines represent an assumed logK_=RSH_|_PM_ constant of 24.0; gray lines represent an assumed logK_=RSH_|_PM_ constant of 22.0. Thin lines represent the modeled contribution of organic = Hg(RS)_2_|_DOM_ to Hg_D_ for the constant H_2_S concentration, a quantity that increases with DOC. Gray bands represent 1–3 mg/L TSS, the threshold below which a majority of Hg in the effluent of nonindustrial wastewater is filter passing.

For decisions about approaches to remove Hg from municipal wastewater, the proportion of Hg present as Hg_P_ and Hg_D_ is of practical concern. For nonindustrial facilities, DOC and Hg_D_ had a relatively small range in the effluent of WRRFs included in this study (Figure 2a,d). Therefore, the quantity of Hg available for removal in the effluent of these facilities can be largely understood in the context of TSS. At TSS concentrations below 1–3 mg/L, Hg_D_ typically comprised a majority of Hg_T_ (Figure 5a) in the facilities involved in this study. Because Hg_D_ was typically between 0.3 and 0.8 ng/L in wastewater effluent, removing TSS to below 1–3 mg/L will also likely lead to compliance with Hg_T_ limits of 2 ng/L or lower. Several facilities with industrial influence that had DOC concentrations exceeding 50 mg/L in effluent wastewater have > 75% Hg_D_ at TSS concentrations higher than 5 mg/L. This shows that, for facilities with industrial sources, Hg bound to DOC could be more abundant than Hg bound to TSS in wastewater effluent. Two of the three observations showing < 60% Hg_D_ at TSS < 1 mg/L were from facilities with membrane treatment that was removing Hg_T_ to well below 2 ng/L.

Complexation modeling shows that the quantity of reduced thiol groups present in DOM and PM of effluent wastewater is abundant enough to preclude the formation of any significant inorganic Hg complexes with OH^−^ and that complexes with HS^−^ are likely to be important only at relatively low (< 5 mg/L) DOC (Figure 5b and Table S3). The assumptions of the model are that the main complexing agents for inorganic Hg in effluent wastewater are reduced thiols in filter‐passing DOM, reduced thiols in particulate PM, and HS^−^ (assumed in the model to be constant at 0.008 mg/L). The results of modeling based on the assumptions and complexation constants from Skyllberg (2008) (*logK*_=RSH_ = 22.0 for thiols in both DOM and PM) predict that a majority of Hg would be in the dissolved phase at TSS concentrations below 30–60 mg/L (Figure 5b, gray lines). This is well above the TSS concentration at which our observations showed a majority of dissolved Hg for nonindustrial samples (3 to 10 mg_TSS_/L, Figure 5a). A larger assumed complexation constant for Hg binding to particulate matter thiols (logK_=RSH_|_PM_ = 24) resulted in a prediction of the TSS at which Hg_D_ was a majority of Hg_T_ that was consistent with observations in wastewater effluent in this study (1–3 mg/L TSS; Figure 5b, black lines). This modeled TSS threshold depends on DOC and simulations with 5, 10, and 50 mg_C_/L are included (Figure 5b). Our model excluded polysulfides and nanocolloidal Hg‐S‐DOM complexes that may be present in wastewater effluent (Pham et al. 2014) but are difficult to describe with thermodynamics. Some of these complexes may be important for carrying filter‐passing Hg in wastewater effluent, but they are not captured by our relatively simple complexation model.

Though the methods of our study are unable to explicitly differentiate between DOM_S_ and PM_S_ that are involved in mercury binding and DOM_S_ and PM_S_ that are not involved in mercury binding, the proportion of sulfur present as strongly Hg‐complexing, thiol‐rich exocyclic reduced sulfur is proportional to the S:C ratio across a range of sulfur content for wetland‐derived natural organic matter (Skyllberg 2008). If the consistent S:C ratio in wastewater effluent contains a similar distribution of sulfur groups to that of natural organic matter, the number of binding sites in wastewater effluent is likely proportional to the quantity of sulfur present in dissolved and particulate form. The fact that wastewater with a variety of and generally low starting S:C ratios for influent PM (Figure S3a) converged on a relatively uniform S:C ratio in effluent material (Figure S3b) suggests a common origin of effluent DOM related to the activated sludge process and could be indicative of a convergence towards consistent characteristics of particulate and dissolved materials involved in binding Hg in wastewater effluent.

## 4 Conclusions

The dominant mechanism of Hg mass removal at municipal WRRFs is Hg binding to organic wastewater particulates during biological treatment followed by the removal of those particles through sedimentation or filtration. However, the ability to meet low‐level mercury limits (e.g., < 2 ng/L) is determined by the very small amount of mercury associated with particles and dissolved organics in the final solids‐control process. The net effect of the activated sludge process, for binding phases relevant to Hg removal, seems to be a transformation and removal of dissolved and particulate organics to conditions with a consistent S:C ratio in wastewater effluent and Hg_D_ of ~0.5 ng/L. This operationally dissolved Hg often became more abundant than particle‐associated Hg when TSS is lower than 1–3 mg/L. A key finding of this study is that low‐level Hg limit compliance is primarily dependent on the effectiveness of TSS removal and not the removal of dissolved organic mercury complexes. For this reason, effective strategies for meeting low‐level Hg limits at WRRFs, especially those without organic‐rich industrial sources, should first prioritize effective particulate removal over dissolved Hg removal.

## Author Contributions

Conceptualization: Nathan W. Johnson and Scott Kyser. Data curation: Nathan W. Johnson, Kelsey Hogan, and Geordee Spilkia. Formal analysis: Nathan W. Johnson, Geordee Spilkia, and Kelsey Hogan. Funding acquisition: Nathan W. Johnson and Scott Kyser. Investigation: Nathan W. Johnson, Adrian T. Hanson, Geordee Spilkia, and Kelsey Hogan. Methodology: Nathan W. Johnson, Adrian T. Hanson, Geordee Spilkia, and Kelsey Hogan. Project administration: Scott Kyser and Nathan W. Johnson. Validation: Nathan W. Johnson, Adrian T. Hanson, and Scott Kyser. Visualization: Nathan W. Johnson and Geordee Spilkia. Writing – original draft: Geordee Spilkia and Nathan W. Johnson. Writing – review and editing: Geordee Spilkia, Nathan W. Johnson, Scott Kyser, Adrian T. Hanson, and Kelsey Hogan.

## Disclosure

The statements, findings, conclusions, and recommendations are those of the authors and do not necessarily reflect the views of the Minnesota Pollution Control Agency.

## Conflicts of Interest

The authors declare no conflicts of interest.

## Supporting information


**Table S1:** Mercury QA/QC data.
**Table S2:** TSS precision by filtered volume.
**Table S3:** Assumed thermodynamic constants for mercury speciation modeling. Calculations for = RSH in PM and DOM.
**Table S4:** Measured *Hg* _*T*_ *(ng/L) and Hg* _*D*_ *(ng/L), TSS [mg/L], DOC, [mg/L] and adjusted SUVA* at the influent, secondary effluent, and tertiary effluent sample locations.
**Table S5:** Surface water total mercury observations in the State of Minnesota lakes and rivers.
**Figure S1:** Schematic of Hg flows in secondary‐ and tertiary‐ treatment for a traditional activated sludge municipal wastewater treatment facility. Stars represent sampling locations. * Samples collected from post‐tertiary locations (before disinfection) only at facilities where tertiary treatment was present.
**Figure S2:** log K_D_ (L/kg) vs. DOC:TSS. Samples from facilities with DOM‐rich industrial influence are indicated with grey dashes. Pond effluent samples are indicated with grey triangles.
**Figure S3:** Sulfur and carbon content (% by mass) of DOM and POM in (a) influent and (b) effluent of municipal wastewater. Lines represent atomic S:C ratios. Samples from plants with DOM‐rich industrial influence are indicated with grey dashes.
**Figure S4:** (a) The relation between DOC‐normalized dissolved Hg in municipal wastewater effluent vs. change (effluent‐influent) in △SUVA. (b) logK_D_ vs. △SUVA between municipal wastewater influent and effluent.
**Figure S5:** Dissolved mercury, as a fraction of total mercury for wastewater influent (squares) and effluent (circles) vs. (a) DOC:TSS ratio and (b) the stoichiometric ratio of dissolved S (mg_S_/L) to particulate S (mg_S_/L). Samples from plants with DOM‐rich industrial influence are indicated with grey dashes. Pond effluent are indicated with grey triangles.

## Data Availability

The data that support the findings of this study are available in the Supporting Information.
