# Supplementary material for: Water Resource Recovery Facilities Meet Low‐Level Mercury Limits by Controlling Effluent Suspended Solids
Source: Water Environ Res. 2025 Aug 6;97(8):e70158. doi: 10.1002/wer.70158 (PMC12328045; doi:10.1002/wer.70158)
Supplement: Supplementary file 1 — Table S1: Mercury QA/QC data. Table S2: TSS precision by filtered volume. Table S3: Assumed thermodynamic constants for mercury speciation modeling. Calculations for = RSH in PM and DOM. Table S4: Measured Hg T (ng/L) and Hg D (ng/L), TSS [mg/L], DOC, [mg/L] and adjusted SUVA at the influent, secondary effluent, and tertiary effluent sample locations. Table S5: Surface water total mercury observations in the State of Minnesota lakes and rivers. Figure S1: Schematic of Hg flows in secondary‐ and tertiary‐ treatment for a traditional activated sludge municipal wastewater treatment facility. Stars represent sampling locations. * Samples collected from post‐tertiary locations (before disinfection) only at facilities where tertiary treatment was present. Figure S2: log KD (L/kg) vs. DOC:TSS. Samples from facilities with DOM‐rich industrial influence are indicated with grey dashes. Pond effluent samples are indicated with grey triangles. Figure S3: Sulfur and carbon content (% by mass) of DOM and POM in (a) influent and (b) effluent of municipal wastewater. Lines represent atomic S:C ratios. Samples from plants with DOM‐rich industrial influence are indicated with grey dashes. Figure S4: (a) The relation between DOC‐normalized dissolved Hg in municipal wastewater effluent vs. change (effluent‐influent) in △SUVA. (b) logKD vs. △SUVA between municipal wastewater influent and effluent. Figure S5: Dissolved mercury, as a fraction of total mercury for wastewater influent (squares) and effluent (circles) vs. (a) DOC:TSS ratio and (b) the stoichiometric ratio of dissolved S (mgS/L) to particulate S (mgS/L). Samples from plants with DOM‐rich industrial influence are indicated with grey dashes. Pond effluent are indicated with grey triangles. [file WER-97-e70158-s001.docx]

`

# Supporting Information

Water resource recovery facilities meet low-level mercury limits by controlling effluent suspended solids

Geordee Spilkia^1^, Scott Kyser^2^, Adrian T. Hanson^1,3^, Kelsey Hogan^3^, Nathan W. Johnson^1,3^

^1^University of Minnesota Water Resources Science Graduate Program,

^2^Minnesota Pollution Control Agency,

^3^University of Minnesota Duluth Department of Civil Engineering

Text S1: Detailed lab methods: DOM isolation, VSS/TSS methods, mercury sample averaging, precision and accuracy

Effluent samples for DOM isolation were filtered and preserved within 48 hours of collection, and influent samples for DOM isolation were filtered within 72 hours of collection. Filtered water for DOM isolation was stored in the dark at 4⁰C until isolation. Bond Elut PPL cartridges (Agilent, 12255002**)** were used to isolate the DOM in 0.45 μm filtered, acidified samples.

Prior to introducing the sample, two cartridge volumes of methanol (v/v; Methanol 99.9%, ACS Grade, Fisher) were pipetted into and drained by gravity through the PPL cartridge followed by five cartridge volumes of MilliQ^®^ water. Filtered (0.45 μm) wastewater samples were pumped into the cartridge using a peristaltic pump with Teflon tubing at a rate below 40 mL/min and not exceeding 2 mmol carbon per g of sorbent. Typical volumes sent through a single cartridge ranged from 500 mL to 4 L, depending on the sample. A composite of the permeate was collected for DOC analysis to determine recovery. Two cartridge volumes of 0.01 M HCl **(**v/v; HCl 37%, Sigma ACS Grade**)** were pumped through the cartridge to remove salts and the permeate was discarded. After 5 minutes of air drying the cartridge, two cartridge volumes of methanol were gravity drained through the cartridge to extract the DOM off the sorbent into combusted glass vials. The methanol-DOM slurry was stored at -20⁰C before blowing down the methanol with nitrogen gas. The sticky DOM isolate was then freeze-dried to remove remaining methanol. Samples were stored at -20⁰C until elemental analysis for C and S. Cartridges were not reused and MilliQ^®^ water blanks were run to ensure there was no contamination from the original cartridges.

For TSS, a glass fiber filter with particulates was dried overnight at 105 °C before weighing. Subsequently, volatile suspended solids (VSS) were quantified by combusting the filters at 550°C for 30 minutes. Volume filtered for TSS and VSS varied among samples and ranged from 5 mL for some influent samples to 2 L for most effluent samples.

The Hg and TSS measurements for independent grab samples were averaged and included as a single value for statistical analysis. Additionally, three to six replicate lab analyses for Hg_T_ and Hg_D_ were performed on a subset of samples, after thorough sample homogenization. The variability in replicate lab analysis (10 to 22 %) was, on average, much lower than the variability in independent grab samples (38 to 53 %, Table S3). The average of replicate lab analyses for a single grab sample was used to calculate the average Hg_T_ and Hg_P_ for the wastewater on a particular date and MWWTP location.

Precision and accuracy were carefully considered since some of the results were close to low-level detection limits for effluent wastewater. Method precision for Hg was calculated using relative differences between duplicate measurements and detection limit of 0.15 ng/L was established. Method precision for DOC and elemental analysis was estimated using the standard deviation of replicate analysis of standards. TSS precision was calculated for each volume of filtered water using error propagation and ranged from 4 to 35 mg/L for most influent samples and 0.1 to 0.4 for most effluent samples (Table S3). The precision of the scale used for TSS measurements is 0.0002 g.

Text S2: Hg speciation calculations

Prior modeling has assumed an average concentration of reduced sulfur in biologically-derived organic matter of 0.5 % by mass of carbon^23^, though the methods employed in this study directly measured the elemental quantity of S. The bulk elemental measurements of C_S,DOM_ and C_S,PM_ were consistent with the quantities of sulfur found in prior measurements of organic matter in natural systems^21^, but cannot explicitly differentiate between S in DOM_S_ and PM_S_ that are involved in Hg binding and S in DOM_S_ and PM_S_ that do not strongly bind Hg^20, 46^. Instead, we used C_S,PM_ and C_S,DOM_ with measurements of carbon in DOM and PM to approximate the abundance of reduced thiol groups in both dissolved and particulate organic matter using the relationship from Poulin et al. 2017: %S_exo_ = 0.021 * (S/C * 1000) + 20 (Supporting Information Text S2)^21, 23^. In our calculations, we first assumed the mercury binding properties of organic thiols in filter-passing (=RSH_DOM_) and particulate (=RSH_PM_) organic matter were identical and also included a stronger binding constant for =RSH_PM_ in an attempt to explain the stoichiometric DOM_S_:PM_S_ ratios at which Hg_D_ comprised a majority of Hg_T_.

The percentage of exocyclic reduced sulfur groups (as a function of total sulfur) was estimated from the S:C ratio of dissolved organic matter (DOM) and particulate matter (PM) according to the relationship developed by Poulin et al. 2017^21^: %Exo = 20 + 0.021 * (atomic S:C * 10^3^). The S:C ratio for PM and DOM in effluent organic matter were consistent at 1 %, leading to a %Exo of 0.41.

An average carbon content for PM (C_C,PM_) of 30% was used to find PM_S_ [umol_S_ / L] from TSS measurements. C_C,PM_ ranged from 19 to 42 % in 6 of 7 samples (one sample had a C_C,PM_ of less than 15). DOM_S_ was estimated from DOC with the consistent 1 % molar S:C ratio for DOM in effluent and adjusted for % exocyclic sulfur.

$${=RSH}_{DOM}\left[ \frac{{mol}_{Sexo}}{L} \right]=\frac{DOC\left[ \frac{{mg}_{C}}{L} \right]}{12000\left[ \frac{{mg}_{C}}{{mol}_{C}} \right]}* atomicS:C\left[ \frac{{mol}_{S}}{{mol}_{C}} \right]* \frac{\%exo}{100}\left[ \frac{{mol}_{Sexo}}{{mol}_{S}} \right]$$

$${=RSH}_{PM}\left[ \frac{{mol}_{Sexo}}{L} \right]=\frac{TSS\left[ \frac{{mg}_{TSS}}{L} \right]*C_{C,PM}\left[ \frac{{mg}_{C}}{{mg}_{TSS}} \right]}{12000\left[ \frac{{mg}_{C}}{{mol}_{C}} \right]}* atomicS:C\left[ \frac{{mol}_{S}}{{mol}_{C}} \right]* \frac{\%exo}{100}\left[ \frac{{mol}_{Sexo}}{{mol}_{S}} \right]$$

The estimated exocyclic reduced thiol quantities were assumed to be the predominant organic sulfur functional groups for speciation modeling in both dissolved and particulate wastewater fractions. The exclusion of HOHgSH^(0)^ as a significant complex in the model amounts to using a logK of -22 rather than -10 for this reaction as recommended by Skyllberg 2008^23^.

The logK value for reduced thiol groups was at first assumed to be the same (22.0) for thiols in both particulate (Table S3, equation 1b) and dissolved organic matter (Table S3, equation 1b’). A larger value of logK_DOM_ (24.0) for equation 1b was also included to reflect the data from this study showing it took a ratio of DOM_S_:PM_S_ much larger than 1 before a majority of Hg was observed in the filter-passing phase (Figure 2b, Figure S5).

With the assumption of a logK value of -22, the influence of dissolved, inorganic H_2_S on the speciation of mercury depends primarily on the two-coordinated sulfur complex (Table S3, equation 12). This complex contributes significantly to filter-passing mercury when DOC is below 2 to 5 mg/L but is much smaller fraction at DOC > 10 mg/L. Calculations were performed at an ΣH_2_S concentration of 0.25 μmol/L, a value that reflects the many measured values that were below the detection limit of 0.25 umol/L in effluent wastewater in this study. Influent wastewater had higher DOC but also higher dissolved sulfide, occasionally up to > 5 μmol/L (Table S4, aqueous sulfide). The thermodynamic modeling was only undertaken for effluent wastewater since the conditions there are most relevant to meeting low-level mercury permit limits.

The fraction of Hg complexed to hydroxide and chloride was insignificant in the organic rich wastewater system. The influence of mercury polysulfides and DOM-stabilized nano-Hg-S complexes, a function of the inorganic sulfur, were not considered in the calculations.

Table S1. Mercury QA/QC data

| **Sample** | **LOD**  **ng/L** | **Recovery of reference material (%)** | **Blanks [ng/L]** | **Relative Percent Difference (lab analysis duplicates)** | **Relative Percent Difference (grab sample replicates)** |
| --- | --- | --- | --- | --- | --- |
| Filtered Hg | 0.15 | 93.7  (n = 34) | 0.1 | 9.8% (n=12) | 41%; n = 25 (effluent)  40%; n = 22 (influent) |
| Total Hg (Unfiltered) |  |  |  | 22% (n = 31) | 38 %; n = 36 (effluent)  53 %; n = 23 (influent) |
|  |  |  |  |  |  |

Table S2: TSS precision by filtered volume.

| Volume (mL) | Precision (mg/L) |
| --- | --- |
| 2000 | 0.1 |
| 1000 | 0.2 |
| 750 | 0.2 |
| 500 | 0.4 |
| 150 | 1.2 |
| 75 | 2.3 |
| 50 | 3.5 |
| 15 | 12 |
| 5 | 35 |

Table S3 Assumed thermodynamic constants for mercury speciation modeling. Calculations for =RSH in PM and DOM.

|  | Reaction | logK | Reference |
| --- | --- | --- | --- |
| 1b | 2RSH_PM_ + Hg^2+^ = Hg(RS)_2,PM_ + 2H^+^ | 22.0  24.0 | Skyllberg 2008^23^  This study |
| 1b’ | 2RSH_DOM_ + Hg^2+^ = Hg(RS)_2,DOM_ + 2H^+^ | 22.0 | Skyllberg 2008^23^ |
| 9 | 2H_2_O + Hg^2+^ = Hg(OH)_2_ + 2H^+^ | -5.2 | Dyrssen and Wedborg [1991]^47^ |
| 12 | 2H_2_S + Hg^2+^ = HgS_2_H^-^ + 3H^+^ | 17.5 | Schwarzenbach and Widmer [1963]^48^ |

Table S3 (continued)

**Supporting Information Table S4.** Measured *Hg_T_ (ng/L) and Hg_D_ (ng/L), TSS [mg/L], DOC, [mg/L] and adjusted SUVA* at the influent, secondary effluent, and tertiary effluent sample locations.

| **Sample Date** | **Facility ID** | **Sample Location** | **Adjusted SUVA** | **DOC (mg/L)** | **TSS (mg/L)** | **Hg_T_ (ng/L)** | **Hg_D_ (ng/L)** |
| --- | --- | --- | --- | --- | --- | --- | --- |
| 6/24/21 | 1 | Influent | 0.71 | 56.09 | 358 | 35.37 | 4.64 |
| 6/23/21 | 2 | Influent | 0.36 | 444.69 | 330 | 34.62 | 3.32 |
| 5/13/21 | 3 | Influent | 1.67 | 13.60 | 93.3 | 14.18 | 3.18 |
| 12/16/2019 | 4 | Influent | 1.64 | 18.16 | 144 | 38.19 | 1.19 |
| 10/25/2019 | 5 | Influent | 0.83 | 32.53 | 99 | 45.02 | 1.96 |
| 10/25/2019 | 6 | Influent | 2.04 | 11.93 | 93 | 9.21 | 0.85 |
| 2/18/2020 | 7 | Influent |  | 196.44 | 600 | 61.36 | 2.96 |
| 5/13/21 | 8 | Influent | 1.10 | 33.92 | 145 | 28.44 | 5.09 |
| 12/19/2019 | 9 | Influent | 0.68 | 55.10 | 158 | 126.79 | 1.68 |
| 10/24/2019 | 10 | Influent | 1.65 | 16.20 | 61 | 3.14 | 1.12 |
| 11/17/2020 | 10 | Influent |  | 75.36 |  | 10.55 | 2.12 |
| 10/24/2019 | 11 | Influent | 1.28 | 12.94 | 26 | 5.01 | 1.63 |
| 9.21.21 | 11 | Influent | 1.93 | 17.64 | 104 | 52.99 | 3.14 |
| 7/20/21 | 12 | Influent | 0.94 | 319.38 | 130 | 18.36 | 5.35 |
| 12/16/2019 | 13 | Influent | 2.89 | 11.52 | 68 | 19.59 | 0.97 |
| 8/3/21 | 13 | Influent | 1.08 | 29.29 | 168 | 30.78 | 0.62 |
| 12/16/2019 | 14 | Influent | 2.11 | 16.43 | 50 | 16.40 | 1.34 |
| 7/8/21 | 14 | Influent | 0.90 | 89.03 | 620 | 209.36 | 9.29 |
| 10/24/2019 | 15 | Influent | 1.22 | 27.97 | 86 | 83.58 | 4.79 |
| 7/8/21 | 15 | Influent | 1.05 | 46.04 | 235 | 19.56 |  |
| 10/24/2019 | 16 | Influent | 0.91 | 62.66 | 214 | 14.16 | 3.68 |
| 9.21.21 | 16 | Influent | 1.57 | 18.69 | 306 | 18.95 | 2.94 |
| 12/16/2019 | 13 | Seconary Effluent | 1.11 | 11.08 | 2 | 2.60 | 0.65 |
| 8/3/21 | 13 | Seconary Effluent | 1.30 | 12.00 | 3.1 | 0.63 | 0.16 |
| 12/16/2019 | 14 | Seconary Effluent | 1.26 | 16.97 | 19 | 4.69 | 0.85 |
| 12/16/2019 | 14 | Seconary Effluent | 1.25 | 17.25 | 19 | 4.92 | 0.54 |
| 7/8/21 | 14 | Seconary Effluent | 1.57 | 12.43 | 9.6 | 0.80 | 0.61 |
| 7/8/21 | 15 | Seconary Effluent | 1.92 | 14.56 | 12.7 | 1.12 | 0.42 |
| 10/24/2019 | 16 | Seconary Effluent | 1.54 | 10.34 | 0.4 | 2.93 | 2.57 |
| 9.21.21 | 16 | Seconary Effluent | 1.84 | 13.20 | 0.7 | 0.85 | 0.61 |
| 6/24/21 | 1 | Secondary effluent | 1.72 | 6.46 | 6.1 | 0.32 | 0.14 |
| 6/23/21 | 2 | Secondary effluent | 1.58 | 8.28 | 8.2 | 0.14 | 0.20 |
| 5/13/21 | 3 | Secondary effluent | 1.45 | 8.97 | 212 | 7.93 | 3.14 |
| 12/16/2019 | 4 | Secondary effluent | 1.29 | 5.08 | 2 | 4.04 | 0.44 |
| 10/25/2019 | 5 | Secondary effluent | 1.58 | 7.82 | 6.8 | 2.68 | 0.58 |
| 10/25/2019 | 6 | Secondary effluent | 2.23 | 8.21 | 5.2 | 1.41 | 0.32 |
| 2/18/2020 | 7 | Secondary effluent |  | 67.92 | 26 | 0.94 | 1.39 |
| 10/24/2019 | 10 | Secondary effluent | 2.16 | 7.52 | 2.7 | 2.79 | 0.29 |
| 10/24/2019 | 11 | Secondary effluent | 1.32 | 7.09 | 52 | 13.20 | 0.53 |
| 9.21.21 | 11 | Secondary effluent | 1.89 | 9.93 | 60 | 10.32 | 1.21 |
| 7/20/21 | 12 | Secondary effluent | 3.41 | 33.99 | 1.6 | 1.59 | 1.51 |
| 12/16/2019 | 14 | Secondary effluent | 2.60 | 14.63 | 17 | 6.69 | 0.58 |
| 7/8/21 | 14 | Secondary effluent | 1.69 | 13.54 | 17 | 1.96 | 0.51 |
| 10/24/2019 | 15 | Secondary effluent | 1.66 | 8.64 | 0.4 | 0.21 | 0.29 |
| 10/24/2019 | 16 | Secondary effluent | 1.54 | 11.66 | 0.6 | 0.27 | 0.53 |
| 6/24/21 | 1 | Tertiary effluent | 1.97 | 6.02 | 2.3 | 0.14 | BDL |
| 6/23/21 | 2 | Tertiary effluent | 1.43 | 8.61 | 0.7 | 0.32 | 0.23 |
| 5/13/21 | 3 | Tertiary effluent | 1.55 | 9.09 | 6 | 4.42 | 4.22 |
| 12/16/2019 | 4 | Tertiary effluent | 1.13 | 6.35 | BDL | 4.77 | 0.39 |
| 10/25/2019 | 5 | Tertiary effluent | 1.74 | 6.97 | 2.5 | 1.19 | 0.58 |
| 10/25/2019 | 6 | Tertiary effluent | 2.21 | 7.89 | 0.4 | 0.86 | 0.90 |
| 2/18/2020 | 7 | Tertiary effluent |  | 61.02 | 11 | 2.24 | 1.76 |
| 5/13/21 | 8 | Tertiary effluent | 1.78 | 6.59 | BDL | 7.00 | 6.80 |
| 12/19/2019 | 9 | Tertiary effluent | 1.81 | 8.71 | BDL | 1.88 | 0.52 |
| 10/24/2019 | 10 | Tertiary effluent | 2.15 | 7.22 | 0.3 | 0.58 | 0.32 |
| 11/17/2020 | 10 | Tertiary effluent |  |  | BDL | 0.59 | 0.55 |
| 10/24/2019 | 11 | Tertiary effluent | 1.66 | 5.96 | 2.5 | 0.94 | 0.45 |
| 9/21/2021 | 11 | Tertiary effluent | 1.52 | 9.12 | 11 | 2.75 | 0.77 |
| 7/20/21 | 12 | Tertiary effluent | 3.31 | 50.80 | 5.6 | 3.17 | 2.55 |

**Supporting Information Table S4 (continued).** Measured *sulfur and carbon content for DOM and POM* in wastewater influent and effluent

a

| **Sample Date** | **Facility ID** | **Sample Location** | **C_C,PM_ (mass %)** | **C_S,PM_ (mass %)** | **C_C,DOM_ (mass %)** | **C_S,DOM_ (mass %)** |
| --- | --- | --- | --- | --- | --- | --- |
| 6/24/21 | 1 | Effluent | BDL | BDL | 52.24 | 2.44 |
| 6/24/21 | 1 | Influent | 44.49 | 0.346 | 52.40 | 2.51 |
| 6/23/21 | 2 | Effluent | BDL | BDL | 49.76 | 1.68 |
| 6/23/21 | 2 | Influent | 40.35 | 0.846 | 47.94 | 1.03 |
| 5/13/21 | 3 | Effluent | 33.15 | 0.944 | 51.56 | 1.32 |
| 5/13/21 | 3 | Influent | 44.22 | 0.399 | 52.14 | 2.00 |
| 5/13/21 | 3 | Post-Sec | 36.88 | 0.573 |  |  |
| 3/23/21 | 4 | Effluent | 33.11 | 0.891 | 50.40 | 1.22 |
| 3/23/21 | 4 | Influent | 43.05 | 0.419 | 50.35 | 2.04 |
| 6/25/20 | 5 | Effluent | 11.46 | 0.051 | 57.11 | 0.89 |
| 6/25/20 | 5 | Influent | 42.70 | 0.41 | 51.51 | 2.25 |
| 10/25/2019 | 6 | Effluent | 44.30 | 1.058 | 52.12 | 2.08 |
| 10/25/2019 | 6 | Influent | 29.35 | 0.473 | 35.81 | 1.41 |
| 6/23/20 | 7 | Effluent | 41.33 | 1.38 | 54.45 | 3.42 |
| 6/23/20 | 7 | Influent | 39.41 | 0.87 | 54.08 | 2.85 |
| 9/24/20 | 7 | Effluent | BDL | BDL | 53.63 | 1.67 |
| 9/24/20 | 7 | Influent |  |  | 52.54 | 2.54 |
| 5/13/21 | 8 | Effluent | BDL | BDL | 51.31 | 1.37 |
| 5/13/21 | 8 | Influent | 42.78 | 0.58 | 51.67 | 2.41 |
| 5/13/21 | 8 | Post-Sec | 32.93 | 0.692 |  |  |
| 6/25/20 | 10 | Effluent | 18.62 | 0.363 | 50.87 | 1.30 |
| 6/25/20 | 10 | Influent | 29.44 | 1.91 | 54.60 | 1.38 |
| 9.21.21 | 11 | Effluent | BDL | BDL | 54.30 | 1.60 |
| 9.21.21 | 11 | Influent | 44.05 | 0.188 | 53.48 | 1.85 |
| 7/20/21 | 12 | Influent | 36.79 | 0.208 | 58.99 | 0.78 |
| 7/20/21 | 12 | Pond 4 |  |  | 48.50 | 1.43 |
| 8/3/21 | 13 | Effluent | BDL | BDL | 52.27 | 1.72 |
| 8/3/21 | 13 | Influent | 42.78 | 0.29 | 54.24 | 2.27 |
| 6/25/2020 | 14 | Effluent | 25.74 | 0.49 | 57.68 | 1.27 |
| 6/25/2020 | 14 | Influent | 31.33 | 0.58 | 54.46 | 1.96 |
| 7/8/21 | 14 | Effluent | BDL | BDL | 53.87 | 1.67 |
| 7/8/21 | 14 | Influent | 43.84 | 0.288 | 51.15 | 1.53 |
| 7/8/21 | 15 | Influent | 40.87 | 0.375 | 54.50 | 3.44 |
| 9.21.21 | 16 | Effluent | BDL | BDL | 51.77 | 1.45 |
| 9.21.21 | 16 | Influent | 55.11 | 0.164 |  |  |

**Supporting Information Table S4 (continued).** Measured *methyl mercury* in selected wastewater samples

| **Date** | **Facility ID** | **Sample Location** | **Sample treatment** | **MeHg [ng/L]** |
| --- | --- | --- | --- | --- |
| 11/17/2020 | 10 | Influent | Unfiltered | 0.83 |
| 11/17/2020 | 10 | Influent | Filtered | 0.75 |
| 11/17/2020 | 10 | Effluent | Unfiltered | 0.03 |
| 11/17/2020 | 10 | Effluent | Filtered | 0.05 |
| 11/17/2020 | 5 | Influent | Unfiltered | 0.72 |
| 11/17/2020 | 5 | Influent | Filtered | 0.11 |
| 11/17/2020 | 5 | Post-Secondary | Unfiltered | 0.05 |
| 11/17/2020 | 5 | Post-Secondary | Filtered | BDL |
| 11/17/2020 | 5 | Effluent | Unfiltered | 0.03 |
| 11/17/2020 | 5 | Effluent | Filtered | 0.03 |
| 2/2/2021 | 5 | Influent | Unfiltered | 0.78 |
| 2/2/2021 | 5 | Influent | Filtered | 0.35 |
| 2/2/2021 | 5 | Post-Secondary | Unfiltered | 0.12 |
| 2/2/2021 | 5 | Post-Secondary | Filtered | 0.05 |
| 2/2/2021 | 5 | Effluent | Unfiltered | BDL |
| 2/2/2021 | 5 | Effluent | Filtered | BDL |
| 3/23/2021 | 6 | Influent | Unfiltered | 0.43 |
| 3/23/2021 | 6 | Influent | Filtered | 0.05 |
| 3/23/2021 | 6 | Post-Secondary | Unfiltered | 0.07 |
| 3/23/2021 | 6 | Effluent | Unfiltered | 0.06 |

**Supporting Information Table S4 (continued).** Measured dissolved sulfide in wastewater influent and effluent sample locations for select WWTP.

| **Date of collection** | **Facility ID** | **Sample Location** | **Filtered/ Unfiltered** | **Sulfide [mg/L]** |
| --- | --- | --- | --- | --- |
| 5/13/2021 | 1 | Effluent | Unfiltered | BDL |
| 5/13/2021 | 2 | Effluent | Unfiltered | BDL |
| 3/23/2021 | 4 | Effluent | Unfiltered | BDL |
| 6/25/2020 | 5 | Effluent | Unfiltered | BDL |
| 2/2/2021 | 5 | Effluent | Unfiltered | BDL |
| 6/25/2020 | 5 (dup) | Effluent | Unfiltered | BDL |
| 3/23/2021 | 6 | Effluent | Unfiltered | BDL |
| 6/25/2020 | 7 | Effluent | Unfiltered | 0.054 |
| 6/25/2020 | 7 (dup) | Effluent | Unfiltered | 0.022 |
| 6/25/2020 | 10 | Effluent | Unfiltered | 0.019 |
| 6/25/2020 | 10 (dup) | Effluent | Unfiltered | 0.038 |
| 6/25/2020 | 10 (dup) | Effluent | Unfiltered | 0.042 |
| 9/21/2021 | 11 | Effluent | Unfiltered | BDL |
| 7/20/2021 | 12 | Effluent | Unfiltered | 0.022 |
| 7/20/2021 | 12 | Effluent | Unfiltered | BDL |
| 8/3/2021 | 13 | Effluent | Unfiltered | BDL |
| 6/25/2020 | 14 | Effluent | Unfiltered | BDL |
| 6/25/2020 | 14 (dup) | Effluent | Unfiltered | 0.029 |
| 7/8/2021 | 14 | Effluent | Unfiltered | 0.019 |
| 9/21/2021 | 16 | Effluent | Unfiltered | BDL |
| 5/13/2021 | 1 | Secondary | Unfiltered | 0.042 |
| 5/13/2021 | 2 | Secondary | Unfiltered | BDL |
| 3/23/2021 | 4 | Secondary | Unfiltered | BDL |
| 2/2/2021 | 5 | Secondary | Unfiltered | BDL |
| 3/23/2021 | 6 | Secondary | Unfiltered | 0.048 |
| 9/21/2021 | 11 | Secondary | Unfiltered | 0.064 |
| 7/20/2021 | 12 | Secondary | Unfiltered | BDL |
| 7/8/2021 | 14 | Secondary | Unfiltered | BDL |
| 5/13/2021 | 1 | Influent | Unfiltered | BDL |
| 3/23/2021 | 4 | Influent | Unfiltered | 0.128 |
| 6/25/2020 | 5 | Influent | Unfiltered | 0.589 |
| 6/25/2020 | 5 (dup) | Influent | Unfiltered | 0.768 |
| 2/2/2021 | 5 | Influent | Unfiltered | 0.157 |
| 3/23/2021 | 6 | Influent | Unfiltered | 0.122 |
| 6/25/2020 | 7 | Influent | Unfiltered | 0.042 |
| 6/25/2020 | 7 (dup) | Influent | Unfiltered | 0.029 |
| 6/25/2020 | 7 | Influent | Filtered | 0.032 |
| 6/25/2020 | 7 (dup) | Influent | Filtered | BDL |
| 6/25/2020 | 10 | Influent | Unfiltered | BDL |
| 9/21/2021 | 11 | Influent | Unfiltered | 0.118 |
| 8/3/2021 | 13 | Influent | Unfiltered | 0.195 |
| 6/25/2020 | 14 | Influent | Unfiltered | 0.032 |
| 6/25/2020 | 14 (dup) | Influent | Unfiltered | BDL |
| 7/8/2021 | 14 | Influent | Unfiltered | 0.349 |
| 7/8/2021 | 15 | Influent | Unfiltered | BDL |
| 9/21/2021 | 16 | Influent | Unfiltered | 0.128 |

**Supporting Information Table S5.** Surface water total mercury observations in the State of Minnesota lakes and rivers.

| **Percentile^1^** | **THg**  **ng/L** |
| --- | --- |
| min | <0.1 |
| 5% | 0.56 |
| 10% | 0.7 |
| 25% | 1.2 |
| 50% | 2.22 |
| 75% | 3.82 |
| 90% | 6.4 |
| 95% | 8.584 |
| max | 33 |
| ^1^ Percentile represents the rank of surface water measurements reported in all regional surface waters over the past 10 years. | |

** only if tertiary treatment was present*

**Supporting Information Figure S1.** Schematic of Hg flows in secondary- and tertiary- treatment for a traditional activated sludge municipal wastewater treatment facility. Stars represent sampling locations. * Samples collected from post-tertiary locations (before disinfection) only at facilities where tertiary treatment was present.


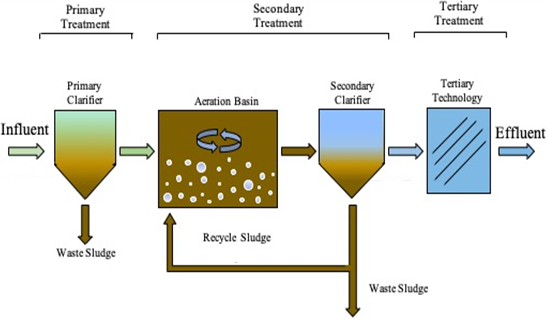

**Supporting Information Figure S2.** log K_D_ (L/kg) vs. DOC:TSS. Samples from facilities with DOM-rich industrial influence are indicated with grey dashes. Pond effluent samples are indicated with grey triangles.

(a)

(b)

(a)

**Supporting Information Figure S3.** Sulfur and carbon content (% by mass) of DOM and POM in (a) influent and (b) effluent of municipal wastewater. Lines represent atomic S:C ratios. Samples from plants with DOM-rich industrial influence are indicated with grey dashes.

(a)

(b)

**Supporting Information Figure S4** (a) The relation between DOC-normalized dissolved Hg in municipal wastewater effluent vs. change (effluent-influent) in ΔSUVA. (b) logK_D_ vs. ΔSUVA between municipal wastewater influent and effluent.

**Supporting Information Figure S5.** Dissolved mercury, as a fraction of total mercury for wastewater influent (squares) and effluent (circles) vs. (a) DOC:TSS ratio and (b) the stoichiometric ratio of dissolved S (mg_S_/L) to particulate S (mg_S_/L). Samples from plants with DOM-rich industrial influence are indicated with grey dashes. Pond effluent are indicated with grey triangles.

(b)

(a)

References

20. A. L.-T. Pham, A. Morris, T. Zhang, J. Ticknor, C. Levard and H. Hsu-Kim, Precipitation of nanoscale mercuric sulfides in the presence of natural organic matter: Structural properties, aggregation, and biotransformation, *Geochimica et Cosmochimica Acta*, 2014, **133**, 204-215.

21. B. A. Poulin, C. A. Gerbig, C. S. Kim, J. P. Stegemeier, J. N. Ryan and G. R. Aiken, Effects of sulfide concentration and dissolved organic matter characteristics on the structure of nanocolloidal metacinnabar, *Environmental Science & Technology*, 2017, **51**, 13133-13142.

23. U. Skyllberg, Competition among thiols and inorganic sulfides and polysulfides for Hg and MeHg in wetland soils and sediments under suboxic conditions: Illumination of controversies and implications for MeHg net production, *Journal of Geophysical Research: Biogeosciences*, 2008, **113**.

46. Y. Wang, J. Liu, V. Liem-Nguyen, S. Tian, S. Zhang, D. Wang and T. Jiang, Binding strength of mercury (II) to different dissolved organic matter: The roles of DOM properties and sources, *Science of The Total Environment*, 2022, **807**, 150979

47. Dyrssen D, Wedborg M. The sulphur-mercury (II) system in natural waters. Water Air & Soil Pollution. 1991 Apr;56:507-19.

48. Schwarzenbach VG, Widmer M. Die löslichkeit von metallsulfiden I. schwarzes quecksilbersulfid. Helvetica chimica acta. 1963;46(7):2613-28.
